# Outbreak of *Salmonella* Bovismorbificans associated with the consumption of uncooked ham products, the Netherlands, 2016 to 2017

**DOI:** 10.2807/1560-7917.ES.2018.23.1.17-00335

**Published:** 2018-01-04

**Authors:** Diederik Brandwagt, Cees van den Wijngaard, Anna Dolores Tulen, Annemieke Christine Mulder, Agnetha Hofhuis, Rianne Jacobs, Max Heck, Anjo Verbruggen, Hans van den Kerkhof, Ife Slegers-Fitz-James, Lapo Mughini-Gras, Eelco Franz

**Affiliations:** 1 Centre for Infectious Disease Control, National Institute for Public Health and the Environment (RIVM), Bilthoven, the Netherlands; 2 European Programme for Intervention Epidemiology Training (EPIET), European Centre for Disease Prevention and Control (ECDC), Stockholm, Sweden; 3 Municipal Health Service (GGD) Utrecht Region, Zeist, the Netherlands; 4 Centre for Nutrition, Prevention and Health Services, National Institute for Public Health and the Environment (RIVM), Bilthoven, the Netherlands; 5 Netherlands Food and Consumer Product Safety Authority (NVWA), Utrecht, the Netherlands

**Keywords:** Salmonella, outbreaks, food-borne infections, epidemiology, trace-back investigation, whole genome sequencing

## Abstract

In January 2017, an increase in reported *Salmonella* *enterica* serotype Bovismorbificans cases in the Netherlands was observed since October 2016. We implemented a case–control study to identify the source, including all cases after December 2016. Adjusted odds ratios were calculated using logistic regression analysis. We traced back the distribution chain of suspected food items and sampled them for microbiological analysis. Human and food isolates were sequenced using whole genome sequencing (WGS). From October 2016 to March 2017, 54 *S.* Bovismorbificans cases were identified. Sequencing indicated that all were infected with identical strains. Twenty-four cases and 37 controls participated in the study. Cases were more likely to have consumed ham products than controls (aOR = 13; 95% CI: 2.0–77) and to have shopped at a supermarket chain (aOR = 7; 95% CI: 1.3–38). Trace-back investigations led to a Belgian meat processor: one retail ham sample originating from this processor tested positive for *S.* Bovismorbificans and matched the outbreak strain by WGS. All ham products related to the same batch were removed from the market to prevent further cases. This investigation illustrates the importance of laboratory surveillance for all *Salmonella* serotypes and the usefulness of WGS in an outbreak investigation.

## Introduction

With ca 27,000 infections per year, salmonellosis is among the most frequent zoonotic infections in the Netherlands [1]. Individual cases of salmonellosis are not notifiable in the Netherlands, except for (para)typhoid fever. The detection of trends in Salmonella serotype distribution therefore depends on the nationwide laboratory surveillance network for gastroenteric pathogens, established in 1987 and covering ca 64% of the population of the Netherlands [2].

### The event

In January 2017, an increase in the number of cases of *Salmonella* Bovismorbificans infections in the Netherlands was reported. From week 41 of 2016 (9 October) until January 2017, 32 *S.* Bovismorbificans cases (one to five per week) were reported to the Center for Disease Control (CIb) of the Dutch National Institute for Public Health and the Environment (RIVM). This number exceeded the expected three to 14 cases of this serotype per year as observed from 2005 to 2015 and was the first possible outbreak of this serotype reported in the Netherlands since the implementation of the laboratory surveillance network [1]. Through an urgent inquiry (UI-393) in the European Centre for Disease Prevention and Control’s Epidemic Intelligence Information System (EPIS), a concurrent increase was reported in Belgium to 32 cases in 2016. An increase in the number of *S*. Bovismorbificans cases was also observed in France where 47 cases were noted in 2016. An outbreak investigation was initiated to find the source of the outbreak and thereby prevent further cases.

## Methods

### Epidemiological investigation

#### Case definition

A case was defined as a person with laboratory-confirmed *S.* Bovismorbificans infection, reported since October 2016, in the Netherlands. This date was selected because the increase in cases was seen since week 41 (starting on 9 October 2016). To generate hypotheses about the source of infection, cases were interviewed using a standardised trawling questionnaire. This questionnaire covered the consumption of different meat products, fish, dairy products, vegetables and fruits, snacks; establishments where food was purchased; if there was contact with a person with diarrhoea and if there was contact with animals during the 7 days before onset of gastro-intestinal symptoms. To address possible recall bias, we only interviewed cases reported since December 2016.

#### Case–control study

As these interviews did not lead to a clear hypothesis, a case–control study was initiated to further explore likely sources. Controls, matched to cases by age, sex and residence municipality, were randomly selected from population registers. Univariable and multivariable odds ratios (OR) and 95% confidence intervals (95% CI) for putative risk factors for *S*. Bovismorbificans infection were calculated using logistic regression analysis. All factors associated in the univariable analysis at p < 0.05 were included in the multivariable analysis based on backward variable selection to yield a model with the most relevant independent risk factors.

Cases who returned the questionnaire were requested to provide additional information on suspected products consumed. This included information on the supermarket chain, producer, product type and European product identification marks of products they bought in the week before symptom onset or that they were buying regularly. This detailed information was shared with the Netherlands Food and Consumer Product Safety Authority (NVWA) to enable them to do a trace-back investigation for suspected food items.

### Whole genome sequencing

In collaboration with the European Centre for Disease Prevention and Control (ECDC), all available Dutch isolates of *S.* Bovismorbificans from human cases in 2016 and those from the other affected countries were sequenced in January 2017, concurrently with the start of the outbreak investigation, using whole genome sequencing (WGS). The Dutch isolates from cases from January to April 2017 were sequenced at a later stage. Sequencing libraries were prepared using Nextera XT chemistry (Illumina Inc., San Diego, United States (US)) for a 250-bp paired-end sequencing run on an Illumina MiSeq sequencer. Samples were sequenced to aim for minimum coverage of 100-fold using Illumina's recommended standard protocols. The resulting FASTQ files de novo were assembled using the SPAdes assembler [3]. Core genome multilocus sequence typing (cgMLST) analysis was conducted using Ridom SeqSphere+ software (version 3.5.0, Ridom GmbH, Münster, Germany) [4]. A Spades assembly of *S.* Bovismorbificans SRR4099590 (http://www.ebi.ac.uk/ena/data/view/SRR4099590) was used as reference to construct a cgMLST gene set by the MLST+ target definer (version 1.1) function of SeqSphere+ with default parameters. Subsequently, the set of outbreak isolates were analysed using this cgMLST scheme and a minimum spanning tree was constructed from the allelic profile using the parameter ‘pairwise ignore missing values’ during distance calculation. One representative human outbreak isolate was uploaded to Enterobase (SAL_KA8933AA) (https://enterobase.warwick.ac.uk/). Persons who were included as a case in the study were excluded from the analysis if sequencing demonstrated the *S.* Bovismorbificans isolated was different (more than five alleles difference) than the outbreak strain.

To have more insight into the presence of the outbreak strain in historical isolates, we searched Enterobase for closely related *S.* Bovismorbificans strains, in both human and non-human isolates. To investigate the presence of the outbreak strain in non-human samples in the Netherlands, we also searched the RIVM surveillance database for all known isolates of *S.* Bovismorbificans in the Netherlands. Relevant strains underwent WGS and were included in the minimum spanning tree.

## Results

### Epidemiological investigation

**Figure 1:**
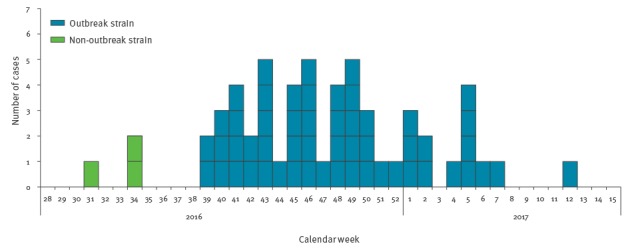
Weekly number of reported cases of *Salmonella* Bovismorbificans by date of symptom onset, the Netherlands, 2016–2017 (n = 54 outbreak cases, n = 3 non-outbreak cases)

From October 2016 to March 2017, 54 cases of *S.* Bovismorbificans were reported in the Netherlands (Figure 1). The cases were 5 to 90 years of age (median 65 years; interquartile range (IQR): 49–73), and 29 were female. The cases were not equally distributed across the country as the majority of them were living in the less densely populated regions in the east and south of the country.

### Case–control study

Twenty-nine cases were reported since December 2016 and consented to participate in the outbreak investigation. Of those, 24 cases responded and participated in the case–control study, together with 37 controls. The day of onset of symptoms for cases varied between 28 November 2016 and 4 February 2017. The cases were 5 to 89 years of age (median 66 years; IQR 45–76) and 14 were female, consistent with the profile of all identified cases. Most frequently reported symptoms were diarrhoea (17 cases) and stomach pain (15 cases), while fever was reported by 11 cases. Fifteen cases were admitted to the hospital due to the severity of their illness, hospitalised cases were 27 to 89 years of age (median 68 years; IQR 43–77).

Food items with the highest frequency of consumption among cases were ham and cheese products. In the univariable analysis, cases were more likely to have consumed raw ham (OR = 12.6; 95% CI: 2.2–125.5) and smoked ham (OR = 5.6; 95% CI: 1.1–36.5) than controls, but were not more likely to have consumed any of the individual cheese products. Furthermore, cases were also more likely to have shopped at a certain supermarket chain (chain 13, see Table). As the participants in both groups did not always remember what specific type of products they had consumed in the incubation period, we merged ham and cheese products into one pooled ham variable (raw, smoked and Coburg ham) and one pooled cheese variable (unsliced, sliced and grated), respectively. In the univariable analysis, cases were more likely to have consumed both ham products (OR = 7.1; 95% CI: 1.9–27.2) and cheese products (OR = 5.6; 95% CI: 1.4–24.7) than controls.

**Table:** Associations between *Salmonella* Bovismorbificans infection and food consumption or purchases at supermarkets, the Netherlands, September 2016 – March 2017.

Food consumption and supermarkets	Cases (n = 24)	Controls (n = 37)	OR	Adjusted OR	95% CI
Ham pooled^a^	15	7	7.1^b^	12.5	2.0–76.6
Cheese pooled^c,d^	20	18	5.6^a^	NA	NA
Supermarket chain 13	17	16	4.5^a^	7.1	1.3–37.9

CI: confidence interval; NA: not applicable; OR: odds ratio.

^a^ Raw, smoked and Coburg ham.

^b^ p value < 0.05.

^c^ Unsliced, sliced and grated cheese.

^d^ Omitted in final model (not significant).

In the multivariable model, we also used the pooled ham and pooled cheese variables. Multivariable analysis confirmed that cases were more likely to have consumed ham products than controls and to have shopped at supermarket chain 13 (Table); consumption of cheese products was not significant and was omitted in the final model.

Based on the results of the case–control study, all cases that were willing to be contacted for further investigation were interviewed again for detailed information on the consumption of ham and cheese products. None of the cases had product leftovers from the exposure period, but three cases provided a photograph of a product as they reported always consuming that same ham product. The identification marks of these products together with supplier information from the suspected supermarket provided some direction in the trace-back investigation.

### Trace-back investigation

The trace-back investigation by the NVWA led to a Belgian meat processor. In cooperation with the Belgian authorities, an inspection was performed by local authorities at the meat processor. No evidence was found for possible contamination during the production process. There were no positive samples of incoming meat and the production of raw ham was completely separated from other meat products to prevent cross contamination. The trace-back investigation in Belgium was not further extended to the farm level. The meat processor intensified its controls after the inspection. The NVWA then decided to focus further investigations on wholesalers and supermarkets in the Netherlands that were supplied by the Belgian meat producer. Samples of both half-finished products and finished (for retail) ham products were taken at several levels in the production chain to be analysed for the presence of *Salmonella*.

In April 2017, 4 weeks after the last case was reported in the Netherlands, one of the collected retail ham products (smoked Coburg ham, sliced at supermarket) tested positive for *Salmonella*. All ham products related to this batch were withdrawn from the Dutch market and the NVWA used the European Rapid Alert System for Food and Feed (RASFF) system to alert the authorities in other European countries. Further serotyping identified the *Salmonella* as serotype Bovismorbificans.

### Whole genome sequencing

**Figure 2:**
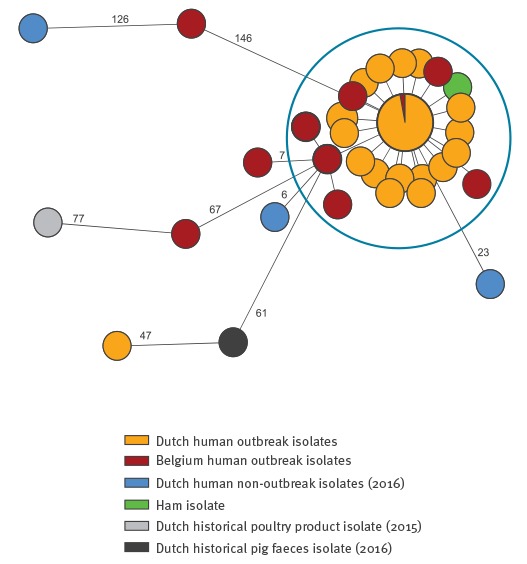
Minimum spanning tree of *Salmonella* Bovismorbificans strains based on core genome multilocus sequence typing analysis, the Netherlands, October 2016–March 2017

Sequencing of human *S.* Bovismorbificans isolates indicated that all isolates taken since October 2016 had less than five alleles difference and were therefore part of the same outbreak. All outbreak strains were ST142 (http://www.genomicepidemiology.org, accessed 18 August 2017). No acquired resistance genes were detected (ResFinder 2.1 http://www.genomicepidemiology.org, accessed 18 August 2017). Outbreak strains possessed the small *Escherichia coli* plasmid pIGJC156 (*col156*, NC_009781, 5,146 bp, 98% homology) with no predicted accessory genes that code for any resistance (PlasmidFinder 1.3, http://www.genomicepidemiology.org, accessed 18 August2017). Next to the Dutch isolates, seven isolates from Belgian patients and two isolates from French patients also matched the outbreak strain. The isolates from other countries and the Dutch patient isolates reported before October 2016 did not match the outbreak strain. WGS indicated that the strain of *S.* Bovismorbificans found in the ham product was identical to the outbreak strain (Figure 2).

The tree was constructed from core-genome MLST allelic profile using the parameter ‘pairwise ignore missing values’ during distance calculation. Numbers correspond to the number of alleles difference between isolates/clusters. Isolates within five alleles difference (i.e. outbreak cluster) are within the blue circle.

In the Enterobase database, 178 hits for *S.* Bovismorbificans ST142 were found with isolation dates from 1970 to 2017. Of those, 26 were non-human isolates. The most closely related strains based on the Enterobase cgMLST V2 were strain SAL_KA7180AA (United Kingdom (UK), unknown source and year of isolation) and SAL_KA4075AA isolated from the Thames River (UK, 2009). In the RIVM surveillance database for the Netherlands, 11 isolates of *S.* Bovismorbificans from non-human sources were recorded. A pig faeces isolate from 2016 and a poultry food product isolate from 2015 (both ST142) underwent WGS. Neither of the strains matched the outbreak strain (Figure 2).

## Discussion

The case–control study identified the consumption of ham products as the vehicle of the outbreak. This finding was confirmed by the identification of *S.* Bovismorbificans in a ham product from a Dutch supermarket, originating from a suspected meat processor, leading to a recall of related ham products to prevent further cases from occurring.

With 57 known cases (54 from the outbreak investigation and three cases that were reported later) over a period of 7 months, this outbreak was one of the smaller outbreaks of *Salmonella* detected in the Netherlands in the past 10 years. With 15 known hospitalisations among 24 interviewed cases, the hospitalisation rate in this outbreak was high compared with the estimated 3% hospitalisations (1,021 hospitalisations of 32,210 salmonella cases) in 2016 for all salmonellosis cases across the country [1]. This suggests the outbreak strain might be more pathogenic than other *Salmonella* strains, thus leading to a higher burden of disease for this specific strain. However, as the number of cases was small, we expect the burden of disease from this outbreak to be limited as the total burden of disease from salmonellosis in the Netherlands is already low compared with some other food-borne pathogens [5,6]. Moreover, the burden of disease estimates of salmonellosis in the Netherlands is based on an overall under-reporting factor of 26 [7]. A more pathogenic strain, which we anticipated was the case in this outbreak, would probably lead to lower rate of under-reporting, suggesting that we may have seen a relatively high number of true cases compared with other *Salmonella* outbreaks.

Smaller and larger outbreaks of *Salmonella* are detected frequently through the Dutch surveillance network, but this was the first outbreak of *S.* Bovismorbificans ever reported in the Netherlands. With three to 14 cases per year (which is less than 1% of all reported cases per year), *S.* Bovismorbificans is one of the less-frequently observed serotypes of *Salmonella* in the Netherlands. This low incidence is comparable with the number of cases reported by other European countries [8]. Such a low background incidence facilitated detection of the increase and the subsequent start of an outbreak investigation. However, since the beginning of the investigation almost no new cases were reported for a period of 4 weeks. With the knowledge that several previous small outbreaks of rare serovars of *Salmonella* had stopped spontaneously, the recruitment of controls was postponed until enough new cases had been reported. Because of this, the start of the analytical study was delayed until the beginning of February.

The investigation led to (uncooked) ham products as the most likely vehicle of transmission in this outbreak. The consumption of raw pork is a known risk factor for salmonellosis; in the Netherlands an estimated 40% of salmonellosis cases are attributed to the consumption of pork [1]. Based on historical data and model-based source attribution analyses, pigs are the primary reservoir of *S.* Bovismorbificans [9]. However, vehicles of transmission detected in outbreaks from Finland, Australia the United States and Germany, include sprouts, hummus and lettuce [10-15]. Only one outbreak of *S.* Bovismorbificans in Germany from 2004 to 2005 was associated with the consumption of pork [16].

Only 15 of 24 cases enrolled in the study reported exposure to ham products in their incubation period. This could be the result of recall bias, as many cases were interviewed 4 to 6 weeks after the day of onset of gastro-intestinal symptoms, i.e. in some cases more than 7 weeks after the exposure. Another explanation could be cross-contamination of other products within individuals’ homes. We assume it unlikely that other food products were involved in the outbreak, as no new cases were reported with the outbreak strain after the withdrawal of the contaminated batch. The low number of positively tested food samples from the incriminated batch of ham suggests that the infectious dose of the contaminated batch was probably not very high. This could also be an explanation for the small number of cases in this outbreak.

WGS indicated that this outbreak was a multi-country outbreak, with a small number of isolates from Belgium and France being identical to the outbreak strain. The number of confirmed cases in these countries was too low to perform epidemiological studies. However, as the trace-back investigation led to a Belgian meat processor, it is likely that contaminated products were also available in Belgian stores and possibly in France.

This outbreak of a rare serotype of *Salmonella*, which led to a recall of suspected ham products, confirms once more the relevance of the Dutch laboratory surveillance system for non-(para)typhi *Salmonella* spp. infections outbreak detection, including that for rare serotypes. In the past 10 years, several outbreaks of *Salmonella* were detected by the system, including a large outbreak of *S.* Thompson in 2012, several smaller outbreaks of *S.* Typhimurium in 2014 and 2015, and a multi-country outbreak of *S.* Enteritidis in 2016 [1,17]. For smaller outbreaks it was not possible to find the source because in some instances the outbreak ended before an outbreak investigation could be initiated or the number of cases was too small to start a case–control study to find an association. In the presented outbreak, the number of cases was also relatively small, but the association with the contaminated food product in the case–control study was strong enough to start a trace-back investigation. The association could have been stronger with a matched analysis design, but the number of discordant pairs was too small.

### Lessons learned

Two key factors in this outbreak investigation were the close collaboration between epidemiologists, microbiologists and food experts of the NVWA from the onset of the outbreak investigation and the use of WGS. Furthermore, the use of a broad trawling questionnaire in the beginning of the outbreak was found to be important. Pork products were not an unexpected source for salmonellosis, but the level of detail in the questionnaire pointed to certain types of ham used as sandwich filling as possible sources of interest already in an early phase.
